# Health System Affiliation and Care for Dual-Eligible and Non–Dual-Eligible Medicare Beneficiaries

**DOI:** 10.1001/jamanetworkopen.2025.38770

**Published:** 2025-10-23

**Authors:** Justin W. Timbie, Maria DeYoreo, Denis Agniel, Shiyuan Zhang, José J. Escarce

**Affiliations:** 1 RAND Corporation, Santa Monica, California; 2 Department of Medicine, David Geffen School of Medicine at the University of California, Los Angeles

## Abstract

**Importance:**

Physician organization (PO) affiliation with health systems and its association with health care disparities for dual-eligible Medicare and Medicaid beneficiaries, who face significant barriers to care, remains underexplored.

**Objectives:**

To estimate the association of health system affiliation with disparities in quality of care and health care utilization for dual-eligible beneficiaries relative to non–dual-eligible Medicare beneficiaries and to decompose these associations into within-PO and between-PO components.

**Design, Setting, and Participants:**

This cohort study used data from 2013 to 2019 on primary care POs and beneficiaries enrolled in traditional Medicare. Statistical analysis was performed from April 2024 to March 2025.

**Exposures:**

Affiliation with health systems, defined as ownership or management relationships, using Medicare Provider Enrollment, Chain, and Ownership System data and Internal Revenue Service Form 990 data.

**Main Outcomes and Measures:**

Eight quality measures assessing receipt of preventive services, chronic condition management, medication adherence, and care coordination and continuity and 5 measures of health care utilization in different settings and with different physician specialists. Linear mixed-effects models were used to estimate changes in disparities before and after affiliation for primary care POs between 2013 and 2019.

**Results:**

A total of 5005 primary care POs and more than 5.6 million Medicare beneficiaries (mean [SD] age, 75.5 [7.5] years; 58.4% women) were analyzed, including approximately 700 000 dual-eligible beneficiaries. Affiliation with health systems was associated with widened disparities in diabetic eye examinations (3.5 percentage point larger relative reduction for dual-eligible beneficiaries compared with non–dual-eligible beneficiaries) and follow-up visits after acute events (3.5 percentage point larger relative reduction). On the other hand, dual-eligible beneficiaries experienced relative improvements in continuity of care with primary care clinicians (1.9 percentage points) and POs (1.4 percentage points) compared with non–dual-eligible beneficiaries, as well as larger relative improvements in statin prescribing (1.8 percentage points), widening preaffiliation differences that favored dual-eligible beneficiaries. Four of these 5 measures were associated with widening within-PO and between-PO disparities. Despite moderate and large preaffiliation disparities in primary care clinician visits and specialist visits, respectively, for dual-eligible beneficiaries relative to non–dual-eligible beneficiaries, disparities in primary care clinician visits widened (21 fewer visits per 100 beneficiaries) while disparities in specialist visits did not change meaningfully.

**Conclusions and Relevance:**

In this cohort study, health system affiliation by primary care POs was associated with both positive and negative associations with disparities for dual-eligible beneficiaries and did not reduce the largest preaffiliation disparities. Health systems must strengthen their care delivery models to expand access to specialists and avoid exacerbating disparities in follow-up care. Health systems could identify factors associated with improved care at high-percentage dual POs for replication.

## Key Points

**Question:**

How is health system affiliation associated with disparities in quality of care and health care utilization between dual-eligible and non–dual-eligible Medicare beneficiaries?

**Findings:**

Using a national sample of primary care physician organizations, this cohort study identified widening disparities for dual-eligible beneficiaries in diabetic eye examinations and follow-up after acute events (3.5 percentage point greater reduction vs non–dual-eligible beneficiaries) after health system affiliation. Although continuity of care and statin prescribing improved, primary care physician visit disparities widened, with 21 fewer visits per 100 beneficiaries.

**Meaning:**

This study suggests that health system affiliation is associated with increased disparities for dual-eligible beneficiaries, highlighting the need for improved strategies to enhance equity and access.

## Teaser

This cohort study uses data on more than 5.6 million Medicare beneficiaries to estimate the association of health system affiliation with disparities in quality of care and health care utilization for dual-eligible Medicare and Medicaid beneficiaries relative to non–dual-eligible beneficiaries.

## Introduction

For the past 2 decades, physician-hospital consolidation has fueled the growth of large health systems—organizations comprising physicians, hospitals, and other facilities that seek to provide the full continuum of care.^1,2,3^ Although health systems might acquire physician organizations (POs) to achieve economies of scale and scope and, potentially, to improve care coordination by aligning operations,^4^ consolidation has also allowed health systems to gain significant leverage with payers,^5^ drive referrals to their hospitals,^6,7^ and block referrals to competitors.^8^ Once employed by health systems, physicians might have less control over the way they provide care,^9^ including whether and how to treat different types of patients, such as patients with high medical needs and low income.

Although prior studies have focused on the association of physician-hospital consolidation with quality of care^10,11,12,13,14^ and prices,^15,16,17^ little is known about the associations of consolidation with health care disparities. Physician organization ownership or management by health systems (hereafter, *affiliation*) could reduce socioeconomic disparities in care if primary care POs that disproportionately serve patients with low income gain greater access to a broad range of specialists who might otherwise limit acceptance of their referrals.^18,19^ Affiliation could also give POs with patients who have high medical needs access to additional staff, such as care coordinators, community health workers, and pharmacists, who can address access barriers and enhance patient engagement in care.^20,21^ At the same time, health systems might reduce care fragmentation by minimizing out-of-system referrals^6,22^ and improve identification of care gaps by deploying advanced quality measurement systems and feedback systems that could reduce disparities within newly affiliating POs.^23^

However, health care disparities could also increase if health system ownership reduces physician autonomy in ways that undermine care for patients with low income. For example, physicians employed by health systems might have less control over their daily schedules, making it more difficult to devote adequate time for patients with complex needs. Compensation for health system physicians, which is often tied to productivity,^24^ might emphasize patient volume over addressing all a patient’s needs. Physicians might also have difficulty advocating for new services or staff who are skilled in serving low-income populations.^25^

We estimated changes in health care disparities for dual-eligible Medicare and Medicaid beneficiaries relative to non–dual-eligible Medicare beneficiaries who received care from independent POs that joined health systems relative to POs that remained independent. Using 13 quality and care utilization measures, we estimated the associations of affiliation overall and then decomposed these associations into changes in within-PO disparities and between-PO disparities. Our study fills an evidence gap in the associations of health system affiliation with care disparities, which, to our knowledge, is limited to a single study focusing on patient adherence to medications.^26^

## Methods

### Identifying POs

For this cohort study, we used the Medicare Data on Provider Practice and Specialty (MD-PPAS) to identify all taxpayer identification numbers (TINs) for which at least 1 physician billed a plurality of Medicare Part B services using the TIN between 2013 and 2019. For 265 of these TINs that were associated with academic health systems, we used crosswalks derived by Welch and Bindman^27^ and updated by our team to combine TINs from the same system, because academic health systems often used multiple TINs corresponding to specialty-specific departments. We used the resulting set of single and combined TINs to represent unique POs. To focus the analysis on primary care POs, we restricted the sample to POs with at least 1 primary care physician and at least 2 total physicians in the baseline year (2013). Primary care was defined by a specialty of general internal medicine, family medicine, geriatric medicine, or general practice. We also required POs to bill Medicare in each year and have at least 30 attributed beneficiaries eligible for a quality or utilization measure in each year (eTable 1 in Supplement 1). This “balanced panel” design minimizes bias from compositional changes due to the entry and exit of POs from the sample, while the denominator size thresholds minimize statistical noise. The RAND Human Subjects Protection Committee approved the study and approved a waiver of informed consent because of the low-risk nature of the study and impracticality of obtaining consent. This study followed the Strengthening the Reporting of Observational Studies in Epidemiology (STROBE) reporting guideline.

### Measuring PO Affiliations With Health Systems

We used data from the Medicare Provider Enrollment, Chain, and Ownership System (PECOS) and Internal Revenue Service (IRS) Form 990 Schedule R from 2013 to 2019 to measure PO affiliations with health systems. We define health systems as entities composed of at least 1 short-term acute care hospital and at least 1 PO that is affiliated through shared ownership or contracting relationships for payment and service delivery. Using PECOS, we defined affiliated POs as those that were either owned or managed by a health system using relationship codes in the data: (1) a 5% or greater direct or indirect ownership interest in the PO or (2) operational or managerial control over the PO whether or not there is an ownership interest by the system. For health systems with 1 or more nonprofit hospitals, we supplemented the PECOS data with IRS Form 990 data by identifying all POs that were listed on a health system’s tax form. For 11 POs that were identified as affiliated in 2 nonconsecutive years but not listed as affiliated in the intervening years, we assumed that the PO was affiliated in each intervening year. See the eMethods in Supplement 1 for more details regarding the identification of new health system affiliations.

### Medicare Beneficiary Attribution

Our sample comprised all Medicare beneficiaries aged 65 years or older enrolled in traditional Medicare whose usual source of care was an eligible PO. We attributed beneficiaries to the PO that provided a plurality of evaluation and management visits to primary care clinicians (PCCs; including physicians, nurse practitioners, and physician assistants) in the calendar year. For beneficiaries without visits to PCCs, we attributed beneficiaries to the PO that provided a plurality of evaluation and management visits to internal medicine subspecialists (see eTable 4 in Supplement 1 for a list of these specialties).

### Measuring PO Characteristics

We used the MD-PPAS to measure the number of unique physicians practicing at each PO and each PO’s mix of physician specialties (ie, primary care only, primary care and other specialties, or only specialties other than primary care). We used Medicare enrollment files to measure the characteristics of beneficiaries attributed to each PO, including their age, sex, race and ethnicity, dual eligibility for Medicare and Medicaid, and original entitlement due to disability. Race and ethnicity were measured using a single variable appearing in the Medicare Beneficiary Summary File with 7 possible options: American Indian or Alaska Native, Asian or Pacific Islander, Black or African American, Hispanic, non-Hispanic White, other [not defined by source data], or unknown). Racial and ethnic differences in quality of care are well documented; we therefore adjusted all analyses for differences in beneficiary race and ethnicity between POs that affiliated and POs that never affiliated. To serve as a proxy for beneficiary comorbidities, we used Hierarchical Condition Category (HCC) scores. Urbanization was measured using common categorizations of Rural-Urban Commuting Area (RUCA) codes.^28^ Each beneficiary’s neighborhood socioeconomic deprivation was measured using a zip code–level index composed of 6 items from the American Community Survey.^29^

### Quality of Care and Utilization Measures

We used 100% Medicare fee-for-service claims files to derive 8 quality measures that reflect the delivery of preventive services (breast cancer screening), chronic condition management (diabetes eye examinations), medication adherence (adherence to blood pressure medications), care coordination (timely follow-up after acute events), and primary care continuity at both the primary care clinician and PO levels. We also measured all-cause hospital readmission rates to assess potential associations with the quality of ambulatory care during care transitions. We also used 5 care utilization measures: primary care clinician visits, internal medicine subspecialist visits, ophthalmologist and optometrist visits, emergency department visits, and a composite measure of ambulatory care–sensitive hospitalizations and ambulatory care–sensitive emergency department visits. eTable 2 in Supplement 1 displays sample sizes and baseline performance, by measure, and eTable 3 in Supplement 1 provides detailed specifications for each measure.

### Statistical Analysis

Statistical analysis was performed from April 2024 to March 2025. We used linear mixed-effects models with covariate centering and random effects for PO and PO-year to estimate the association of affiliation with disparities between dual-eligible and non–dual-eligible beneficiaries between 2013 and 2019 and to decompose the overall association of affiliation into associations with both within-PO disparities and between-PO disparities. Association estimates were measured in the first full year after the year of affiliation through 2019. Within-PO disparities refer to differences in quality of care or utilization between dual-eligible and non–dual-eligible beneficiaries within the same PO, reflecting potential inequities in care for patients served by the same organization. By contrast, between-PO disparities capture differences in outcomes across POs that have varying proportions of dual-eligible patients, indicating whether organizations that disproportionately serve populations with high medical needs achieve different levels of quality or care utilization overall. Estimates of both types of disparities can inform policy questions about whether disparities are associated more with unequal treatment within organizations vs systematic differences across organizations, helping target interventions either at the organization or system level to improve health equity. The eMethods in Supplement 1 contains a description of the model, a more detailed version of which has been published elsewhere.^30^

The model was adjusted for beneficiary age, sex, original Medicare entitlement due to disability, race and ethnicity, HCC score, RUCA category, and area-level socioeconomic deprivation. All models included adjustment for the following time-varying PO characteristics: number of attributed beneficiaries, number of physicians, specialty mix (primary care only vs primary care and other specialties), mean age of beneficiary, mean risk score, mean area-level deprivation, percentage of disabled beneficiaries, percentage of female beneficiaries, percentage of beneficiaries in each RUCA category, and percentage of beneficiaries by race and ethnicity. Inclusion of these characteristics helps to control for changes in patient mix and PO attributes unrelated to affiliation that may be associated with changes in quality and care utilization and is consistent with our assumption of conditional parallel trends. We used an event-study design^31,32,33^ to confirm the presence of conditional parallel trends in within-PO disparities in the preaffiliation period between POs that were never affiliated and POs that became affiliated later in the study period for all measures included in the analysis (eTable 4 in Supplement 1). All estimates associated with all figures are included in eTables 5 to 8 in Supplement 1. Analyses were conducted using R, version 4.4.1 (R Project for Statistical Computing).

## Results

A total of 5005 primary care POs and more than 5.6 million Medicare beneficiaries (mean [SD] age, 75.5 [7.5] years; 58.4% women and 41.6% men; 1.7% Asian or Pacific Islander beneficiaries, 6.8% Black beneficiaries, 4.6% Hispanic beneficiaries, 84.9% White beneficiaries, and 1.3% beneficiaries of other race or ethnicity) per year were included in the analysis, including approximately 700 000 dual-eligible beneficiaries. Physician organizations that affiliated with health systems during the study period were, on average, larger than nonaffiliating POs (mean [SD], 22.2 [53.0] vs 13.7 [87.5] physicians; 1804.8 [2642.5] vs 1110.4 [2028.2] beneficiaries), and they were more likely to include physicians with specialties other than primary care (50.7% vs 33.5%) (Table). Beneficiaries attributed to affiliating POs were less likely to be dually eligible for Medicare and Medicaid (9.4% vs 12.8%) and more likely to be White (89.0% vs 84.2%) and less likely to be Asian or Pacific Islander (1.2% vs 1.8%), Black (5.7% vs 7.0%), Hispanic (2.6% vs 4.9%), or from other racial and ethnic groups (0.8% vs 1.4%). Beneficiaries attributed to the 2 types of POs were similar with respect to all other sociodemographic characteristics and HCC scores. Comparisons of dual-eligible and non–dual-eligible beneficiaries in both types of POs are included in eTable 9 in Supplement 1.

**Table.** Beneficiary and Physician Organization Characteristics

Characteristic^a^	Affiliating physician organizations (n = 446)	Nonaffiliating physician organizations (n = 4559)
**Beneficiary-level characteristics, patients, No./total No. (%)**		
Age, mean (SD), y	75.8 (7.6)	75.5 (7.5)
Sex		
Female	457 639/780 112 (58.7)	2 844 235/4 870 787 (58.4)
Male	322 473/780 112 (41.3)	2 026 552/4 870 787 (41.6)
Dual eligibility	72 987/780 112 (9.4)	625 775/4 870 787 (12.8)
Disabled	65 248/780 112 (8.4)	444 549/4 870 787 (9.1)
Race and ethnicity		
Asian or Pacific Islander	9575/780 112 (1.2)	88 967/4 870 787 (1.8)
Black	44 259/780 112 (5.7)	339 420/4 870 787 (7.0)
Hispanic	20 527/780 112 (2.6)	236 779/4 870 787 (4.9)
White	694 448/780 112 (89.0)	4 102 386/4 870 787 (84.2)
Other^b^	5993/780 112 (0.8)	69 718/4 870 787 (1.4)
Urbanicity		
Metropolitan RUCA	604 156/780 112 (77.4)	3 790 196/4 870 787 (77.8)
Micropolitan RUCA	94 820/780 112 (12.2)	596 812/4 870 787 (12.3)
Small-town RUCA	49 485/780 112 (6.3)	286 825/4 870 787 (5.9)
Rural RUCA	31 011/780 112 (4.0)	193 637/4 870 787 (4.0)
HCC score, mean (SD)	1.1 (1.0)	1.1 (1.0)
Area-level SES index, mean (SD)^c^	0.1 (0.9)	0.0 (0.9)
**Physician organization–level characteristics**		
No. of beneficiaries, mean (SD)	1804.8 (2642.5)	1110.4 (2028.2)
No. of physicians, mean (SD)	22.2 (53.0)	13.7 (87.5)
Specialty mix, No./total No. (%)		
Primary care only	220/446 (49.3)	3031/4559 (66.5)
Primary care and other specialties	226/446 (50.7)	1528/4559 (33.5)

Abbreviations: HCC, Hierarchical Condition Category; RUCA, Rural-Urban Commuting Area; SES, socioeconomic status.

^a^ Characteristics of 4268 physician organizations meeting all inclusion criteria for the analysis are displayed in the table and reflect values in the baseline year (2013).

^b^ This group includes American Indian or Alaska Native beneficiaries and beneficiaries with other race (“other” not defined by source data) listed in the Medicare Beneficiary Summary File.

^c^ The area-level SES index includes 6 items: percentage graduating high school, percentage male unemployment, percentage of households living below the federal poverty level, percentage of female-headed households with children, percentage of households receiving public assistance, and median annual household income. Higher scores indicate higher socioeconomic status.

### Overall Association of Affiliation With Disparities

**Figure 1.**
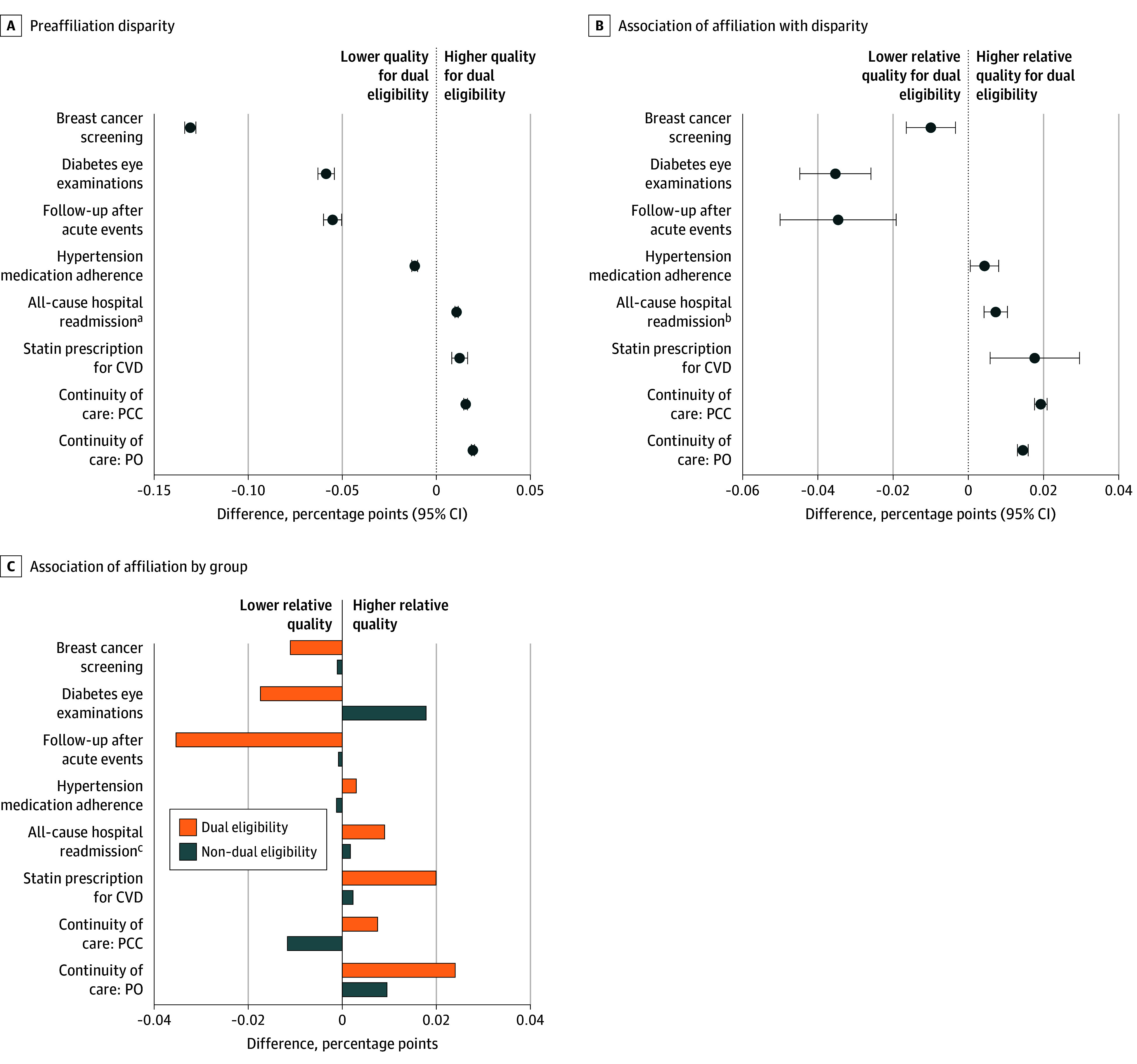
Overall Disparities in Quality of Care Between Dual-Eligible and Non–Dual-Eligible Beneficiaries Before Affiliation and Association of Affiliation With Disparities All estimates associated with the forest plots are included in eTable 5 in Supplement 1. CVD indicates cardiovascular disease; PCC, primary care clinician; and PO, physician organization. ^a^Positive values indicate lower quality for dual eligibility. ^b^Positive values indicate lower relative quality for dual eligibility. ^c^Positive values indicate lower relative quality.

Preaffiliation disparities in quality of care for dual-eligible beneficiaries relative to non–dual-eligible beneficiaries were large for several measures, including a 13.1 (95% CI, 12.8-13.4) percentage point lower rate of breast cancer screening, a 5.9 (95% CI, 5.4-6.3) percentage point lower rate of diabetic eye examinations, and a 5.5 (95% CI, 5.0-6.0) percentage point lower rate of follow-up visits after acute events (Figure 1). Affiliation with health systems was associated with widened disparities in diabetic eye examinations by 3.5 (95% CI, 2.6-4.5) percentage points and with follow-up visits after acute events by 3.5 (95% CI, 1.9-5.0) percentage points due to reductions in quality for dual-eligible beneficiaries and either no change or improved care for non–dual-eligible beneficiaries. Meanwhile, dual-eligible beneficiaries experienced relative improvements in continuity of care with primary care clinicians (1.9 [95% CI, 1.8-2.1] percentage points) and POs (1.4 [95% CI, 1.3-1.6] percentage points) compared with non–dual-eligible beneficiaries, as well as larger relative improvements in statin prescribing (1.8 [95% CI, 0.6-3.0] percentage points), widening preaffiliation differences that favored dual-eligible beneficiaries for each of these 3 measures.

**Figure 2.**
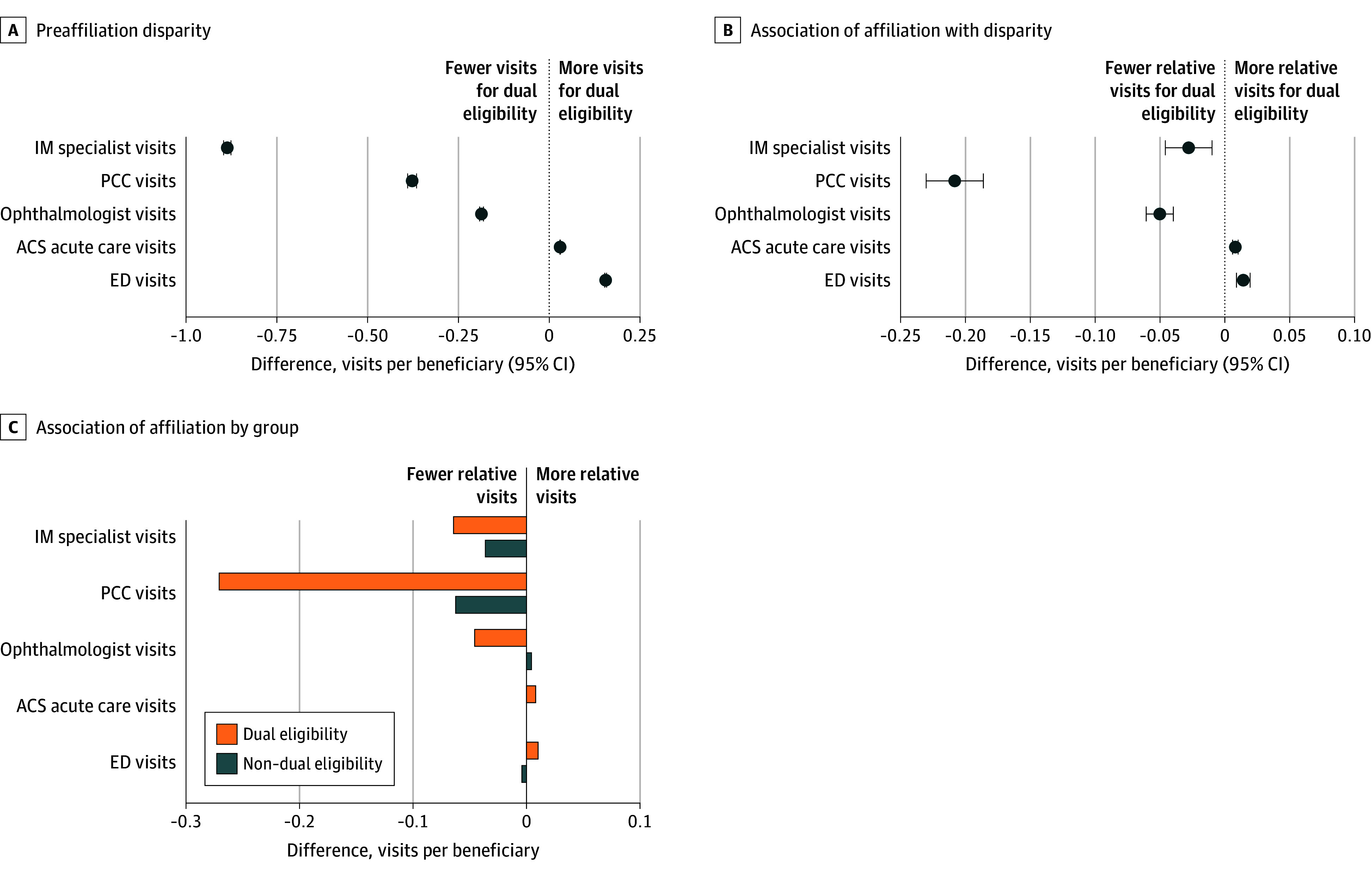
Overall Disparities in Health Care Utilization Between Dual-Eligible and Non–Dual-Eligible Beneficiaries Before Affiliation and Association of Affiliation With Disparities All estimates associated with the forest plots are included in eTable 6 in Supplement 1. ACS indicates ambulatory care–sensitive; ED, emergency department; IM, internal medicine; and PCC, primary care clinician.

Dual-eligible beneficiaries had fewer visits with specialists than non–dual-eligible beneficiaries for all 3 categories of specialists examined before affiliation, including 89 (95% CI, 88-90) fewer visits to internal medicine subspecialists for every 100 beneficiaries and 38 (95% CI, 37-39) fewer visits to PCCs; they also had higher rates of emergency department visits (Figure 2). Affiliation was associated with widening each of these disparities slightly except for PCC visits, for which the disparity increased by nearly 50%—a relative reduction of 21 (95% CI, 19-23) PCC visits per 100 beneficiaries after affiliation.

### Decomposed Associations of Affiliation With Quality of Care

**Figure 3.**
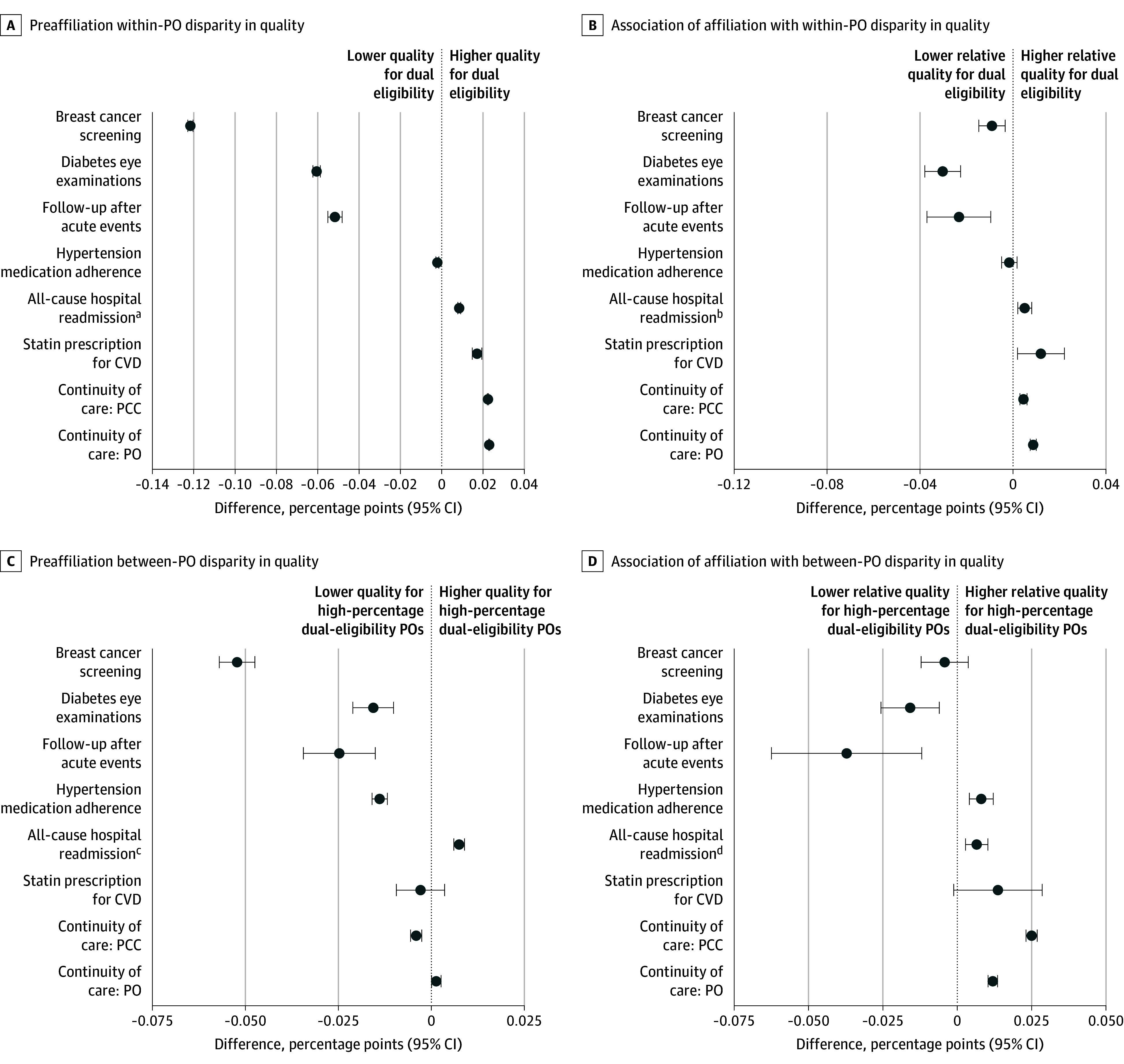
Within–Physician Organization (PO) and Between-PO Disparities in Quality of Care Between Dual-Eligible and Non–Dual-Eligible Beneficiaries Before Affiliation and Association of Affiliation With Disparities Between-PO disparities are estimated as differences in quality between POs with a high percentage of dual-eligible beneficiaries (defined as the 80th percentile of the PO distribution [35.8%]) relative to POs with a low percentage (defined as the 20th percentile of the PO distribution [7.2%]). All estimates associated with the forest plots are included in eTable 7 in Supplement 1. CVD indicates cardiovascular disease; and PCC, primary care clinician. ^a^Positive values indicate lower quality for dual eligibility. ^b^Positive values indicate lower relative quality for dual eligibility. ^c^Positive values indicate lower relative quality for high-percentage dual-eligibility POs. ^d^Positive values indicate lower relative quality for high-percentage dual-eligibility POs.

The widening disparities in diabetic eye examinations and follow-up after acute events were associated with both wider within-PO disparities and larger reductions in quality for high-percentage dual POs relative to low-percentage dual POs (Figure 3). For example, rates of follow-up visits after acute events decreased 2.3 (95% CI, 1.0-3.7) percentage points more for dual-eligible beneficiaries than for non–dual-eligible beneficiaries within POs, and they decreased 3.7 (95% CI, 1.2-6.3) percentage points more for high-percentage dual POs than low-percentage dual POs. Changes in diabetic eye examinations followed a similar pattern, although the within-PO associations were slightly larger (3.0 [95% CI, 2.3-3.8] percentage point larger reduction in quality for dual-eligible beneficiaries), while the between-PO associations were smaller (1.6 [95% CI, 0.6-2.6] percentage point larger relative reduction in quality for high-percentage dual POs). By contrast, affiliation was associated with a larger relative improvement in continuity of primary care with individual PCCs in high-percentage dual POs compared with low-percentage dual POs, but the corresponding change in within-PO disparities was negligible. (The eFigure in Supplement 1 provides more detailed results describing the direction of the association of affiliation for each measure.)

### Decomposed Associations of Affiliation With Care Utilization Measures

**Figure 4.**
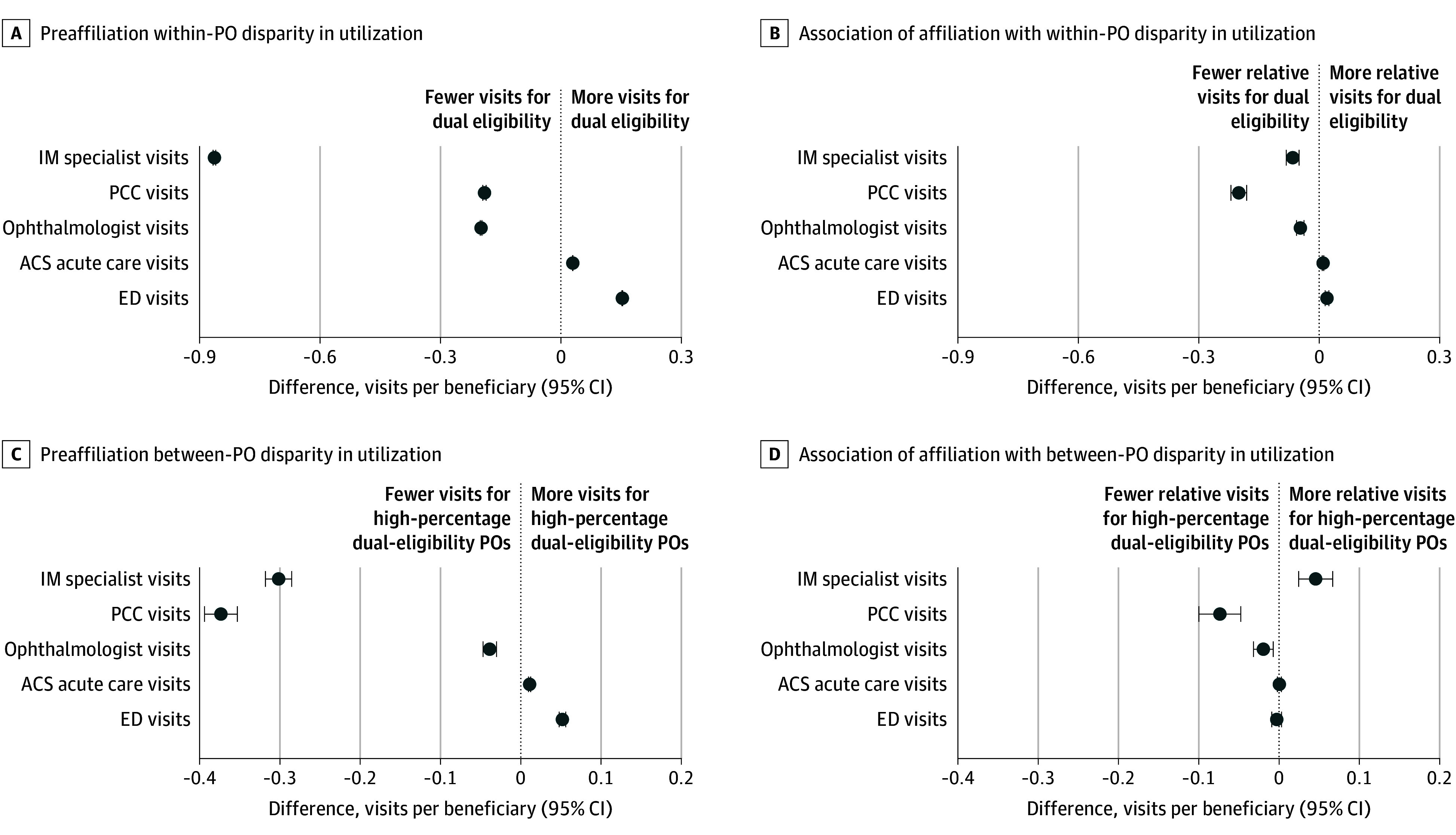
Within–Physician Organization (PO) and Between-PO Disparities in Health Care Utilization Between Dual-Eligible and Non–Dual-Eligible Beneficiaries Before Affiliation and Association of Affiliation With Disparities Between-PO disparities are estimated as differences in utilization between POs with a high percentage of dual-eligible beneficiaries (defined as the 80th percentile of the PO distribution [35.8%]) relative to POs with a low percentage (defined as the 20th percentile of the PO distribution [7.2%]). All estimates associated with the forest plots are included in eTable 8 in Supplement 1. ACS indicates ambulatory care–sensitive; ED, emergency department; IM, internal medicine; and PCC, primary care clinician.

The larger relative reduction in PCC visits for dual-eligible beneficiaries after affiliation was associated with both widening within-PO disparities (20 [95% CI, 18-22] fewer visits per 100 beneficiaries than non–dual-eligible beneficiaries within the same PO) and wider between-PO disparities (7 [95% CI, 5-10] fewer visits per 100 beneficiaries in high-percentage dual POs than low-percentage dual POs) (Figure 4). Although high-percentage dual POs were associated with a relative increase in specialist visits (5 [95% CI, 2-7] additional visits per 100 beneficiaries compared with low-percentage dual POs), the widening within-PO disparity (7 [95% CI, 5-8] fewer visits per 100 beneficiaries for dual-eligible beneficiaries than non–dual-eligible beneficiaries within the same PO) contributed to the widening disparity for dual-eligible beneficiaries overall.

## Discussion

Few studies have assessed the association of physician-hospital consolidation with populations with high needs who face barriers accessing care. In our analysis that followed primary care POs for a period of up to 5 years after joining health systems, we found significant disparities in quality and health care utilization between dual-eligible and non–dual-eligible beneficiaries before affiliation, and many of these disparities either widened or remained largely unchanged after affiliation. Health system affiliation was associated with large reductions in quality of care for dual-eligible beneficiaries across multiple measures, including a 3.5 percentage point relative reduction in rates of follow-up visits after acute events and diabetic eye examinations compared with non–dual-eligible beneficiaries. However, dual-eligible beneficiaries also experienced larger improvements in continuity of care and statin prescribing than non–dual-eligible beneficiaries. Disparities in PCC visits for dual-eligible beneficiaries widened substantially, while the large preaffiliation disparities in specialist visits changed little after affiliation.

Although prior studies have identified modest benefits of health system affiliation associated with quality of care for patients overall,^10,14^ we found that patients with high needs may be at risk of experiencing reductions in quality across multiple measures. Although health systems can reduce care fragmentation through common electronic health record platforms, improve standardization through systemwide care protocols, and address disparities using advanced quality measurements and improvement infrastructure,^34,35^ newly affiliating POs might not be adopting this infrastructure, or these changes might not lead to improved care over a 1- to 5-year period. At the same time, patients might elect to switch to affiliated specialists once their PCCs become affiliated with these specialists (or patients might be encouraged to switch by their health systems), which would require patients to develop new relationships and build trust with these PCCs and might be burdensome for patients with high needs, thereby explaining the reductions in diabetic eye examinations we observed. Health systems, which are more likely than independent physician practices to use telehealth,^36^ might conduct a higher proportion of follow-up visits by telemedicine rather than in person to improve scheduling efficiency.

The widening disparities in quality measures might also be due to poorer access for dual-eligible beneficiaries and are consistent with our finding of relative reductions in visits with PCCs. Reductions in visits could be due to new appointment scheduling processes or policies, including the introduction of late policies or no-show policies (although there are limited empirical data on differences in use of these policies across PO models), or use of call centers or other processes that make it harder for patients with low income and complex conditions to access care.^37^ Alternatively, the reductions in PCC visits could indicate more efficient delivery of primary care services. Similarly, while reductions in specialist visits for dual-eligible beneficiaries could theoretically be due to more efficient care processes, such as greater use of e-consults,^38^ other studies have shown that dual-eligible beneficiaries have historically lacked access to specialty care,^39^ and there is limited evidence of overuse of specialty care.

Continuity of care is a cornerstone of advanced primary care that can foster a trusting relationship between patients and PCCs.^40^ We found that continuity with both individual PCCs and POs improved more for dual-eligible beneficiaries than non–dual-eligible beneficiaries after health system affiliation. This finding suggests that health systems might more systematically seek to empanel patients with high need on care teams or that newly affiliating POs might gain access to more services or staff who can support delivery of a more comprehensive set of primary care services. At the same time, health systems may be more likely than independent practices to attempt to keep all care for dual-eligible beneficiaries within the system.

Despite evidence of widening disparities for dual-eligible beneficiaries in primary care POs that joined health systems, affiliation may nevertheless be associated with significant benefits to patients, particularly patients with complex conditions who require highly specialized care teams that coordinate closely to address multiple health conditions. These benefits may not be fully apparent in comparisons of care between dual-eligible and non–dual-eligible Medicare beneficiaries, and they may not be captured in quality measures reflecting delivery of primary care services. Nevertheless, our results suggest that the tradeoff required of patients to achieve these benefits might be reduced access to primary care that may, in turn, undermine the management of chronic conditions. Further understanding the extent to which the widening disparities we observed are associated with health system–driven care processes or patients’ abilities to adapt to new methods of accessing care and communicating with PCCs after health system affiliation are priorities for future work.

### Limitations

Our findings should be considered in light of several limitations. First, some independent POs might be erroneously linked with health systems and vice versa as there is no single-criterion standard source for this information. Although PECOS in combination with IRS data have been used in multiple prior studies,^41,42^ recent research suggests that combining additional data sources can more completely capture these associations.^43^ Second, affiliations with health systems could include a variety of relationships between physicians and health systems, including employment, contractual relationships, or other types of partnerships, that may be associated with heterogeneous associations with disparities, but the lack of data on these relationships prevented us from exploring these associations. Third, our analysis is limited to POs that join a health system identified by both a change in ownership or management information in PECOS and a change of TIN. Although we used this more stringent definition to reduce the chance of measurement error in the PECOS data, it might omit some new affiliations. Fourth, our analysis focuses on beneficiaries enrolled in traditional Medicare, which may limit the generalizability of findings. However, Medicare Advantage encounter data were not available for the earliest years of our study period, and many have expressed concerns about the completeness of the Medicare Advantage encounter data in its first few years.^44^ Fifth, our analysis did not focus on beneficiary switching patterns during the year of affiliation or subsequent years or the extent to which associations of affiliation might be stronger after a transition period of 1 or more years. Sixth, we estimated the association of health system affiliation with measures that can be derived from claims alone. A comprehensive assessment would incorporate beneficiary care experiences as well as measures of chronic condition management, such as hemoglobin A_1c_ and blood pressure control for patients with diabetes, and information about access to and use of enabling services that could strengthen patient care, such as health coaches and care managers.

## Conclusions

In this cohort study, we found that health system affiliation was associated with both wider disparities and a lack of improvement in reducing existing disparities across multiple measures of quality and health care utilization. Despite recent studies suggesting that health system–affiliated physicians might provide slightly higher quality of care, our findings suggest that dual-eligible beneficiaries received a lower quality of care across multiple measures—possibly through reduced access to primary care—although these beneficiaries also appeared to experience improvements in continuity of care and statin prescribing. Future research should explore the mechanisms driving these disparities and examine the broader implications for other vulnerable populations.

## Notes

Supplement 1. **eTable 1.** Derivation of Physician Organization and Medicare Beneficiary Sample **eTable 2.** Measure-Specific Sample Sizes and Baseline Performance **eTable 3.** Quality and Utilization Measure Specifications **eTable 4.** Parallel Trends Assessment **eTable 5.** Overall Disparities in Quality of Care Between Dual-Eligible and Non–Dual-Eligible Beneficiaries Before Affiliation and Impact of Affiliation (Figure 1 in Manuscript) **eTable 6.** Overall Disparities in Utilization Between Dual-Eligible and Non–Dual-Eligible Beneficiaries Before Affiliation and Impact of Affiliation (Figure 2 in Manuscript) **eTable 7.** Within-PO and Between-PO Disparities in Quality of Care Between Dual-Eligible and Non–Dual-Eligible Beneficiaries Before Affiliation and Impact of Affiliation (Figure 3 in Manuscript) **eTable 8.** Within-PO and Between-PO Disparities in Utilization Between Dual-Eligible and Non–Dual-Eligible Beneficiaries Before Affiliation and Impact of Affiliation (Figure 4 in Manuscript) **eTable 9.** Comparisons of Beneficiary Characteristics by Dual-Eligibility Status and Attributed PO Type **eFigure.** Impact of Health System Affiliation for Dual-Eligible and Non–Dual-Eligible Beneficiaries Who Receive Care From POs With a Low or High Percentage of Dual-Eligible Beneficiaries
eMethods.

eReferences.

Supplement 2.
Data Sharing Statement
