# Supplementary material for: Health System Affiliation and Care for Dual-Eligible and Non–Dual-Eligible Medicare Beneficiaries
Source: JAMA Netw Open. 2025 Oct 23;8(10):e2538770. doi: 10.1001/jamanetworkopen.2025.38770 (PMC12550639; doi:10.1001/jamanetworkopen.2025.38770)
Supplement: Supplement 1. — eTable 1. Derivation of Physician Organization and Medicare Beneficiary Sample eTable 2. Measure-Specific Sample Sizes and Baseline Performance eTable 3. Quality and Utilization Measure Specifications eTable 4. Parallel Trends Assessment eTable 5. Overall Disparities in Quality of Care Between Dual-Eligible and Non–Dual-Eligible Beneficiaries Before Affiliation and Impact of Affiliation (Figure 1 in Manuscript) eTable 6. Overall Disparities in Utilization Between Dual-Eligible and Non–Dual-Eligible Beneficiaries Before Affiliation and Impact of Affiliation (Figure 2 in Manuscript) eTable 7. Within-PO and Between-PO Disparities in Quality of Care Between Dual-Eligible and Non–Dual-Eligible Beneficiaries Before Affiliation and Impact of Affiliation (Figure 3 in Manuscript) eTable 8. Within-PO and Between-PO Disparities in Utilization Between Dual-Eligible and Non–Dual-Eligible Beneficiaries Before Affiliation and Impact of Affiliation (Figure 4 in Manuscript) eTable 9. Comparisons of Beneficiary Characteristics by Dual-Eligibility Status and Attributed PO Type eFigure. Impact of Health System Affiliation for Dual-Eligible and Non–Dual-Eligible Beneficiaries Who Receive Care From POs With a Low or High Percentage of Dual-Eligible Beneficiaries eMethods. eReferences. [file jamanetwopen-e2538770-s001.pdf]

Supplementary Online Content

Timbie JW, DeYoreo M, Agniel D, Zhang S, Escarce JJ. Health system affiliation and care for dual-eligible and non–dual-eligible Medicare beneficiaries. *JAMA Netw Open.* 2025;8(10):e2538770. doi:10.1001/jamanetworkopen.2025.38770

eTable 1. Derivation of Physician Organization and Medicare Beneficiary Sample
eTable 2. Measure-Specific Sample Sizes and Baseline Performance
eTable 3. Quality and Utilization Measure Specifications
eTable 4. Parallel Trends Assessment
eTable 5. Overall Disparities in Quality of Care Between Dual-Eligible and Non–Dual-Eligible Beneficiaries Before Affiliation and Impact of Affiliation (Figure 1 in Manuscript)
eTable 6. Overall Disparities in Utilization Between Dual-Eligible and Non–Dual-Eligible Beneficiaries Before Affiliation and Impact of Affiliation (Figure 2 in Manuscript)
eTable 7. Within-PO and Between-PO Disparities in Quality of Care Between Dual-Eligible and Non–Dual-Eligible Beneficiaries Before Affiliation and Impact of Affiliation (Figure 3 in Manuscript)
eTable 8. Within-PO and Between-PO Disparities in Utilization Between Dual-Eligible and Non–Dual-Eligible Beneficiaries Before Affiliation and Impact of Affiliation (Figure 4 in Manuscript)
eTable 9. Comparisons of Beneficiary Characteristics by Dual-Eligibility Status and Attributed PO Type
eFigure. Impact of Health System Affiliation for Dual-Eligible and Non–Dual-Eligible Beneficiaries Who Receive Care From POs With a Low or High Percentage of Dual-Eligible Beneficiaries
eMethods.
eReferences.

This supplementary material has been provided by the authors to give readers additional information about their work.

© 2025 Timbie JW et al. *JAMA Network Open.*

eTable 1. Derivation of Physician Organization and Medicare Beneficiary Sample

	Physician Organizations		Medicare Beneficiaries	
Sample inclusion/exclusion criterion	#	%	#	%
1. Unaffiliated POs in 2013 with >=2 physicians, >=1 primary care physician, >=1 attributed beneficiary, and TIN is present in MDPPAS for all 7 years	9,275	100	7,467,234	100
2. Exclude POs that both affiliate and de-affiliate	9,099	98.1	7,076,340	94.8
3. Exclude POs that have <=30 duals and <=30 non-duals in each study year	5,005	54.0	5,650,899	75.7

© 2025 Timbie JW et al. *JAMA Network Open.*

eTable 2. Measure-Specific Sample Sizes and Baseline Performance

	Becomes Affiliated[a]			Never Affiliated		
Measure	# POs	# Beneficiaries	Baseline performance (%)	# POs	# Beneficiaries	Baseline performance (%)
**Quality measures**						
All-Cause Hospital Readmissions	311	122,006	15.7	2,788	723,408	15.7
Breast Cancer Screening	176	101,798	72.5	1,485	621,106	70.2
Continuity of Care – PCC	351	460,022	76.7	3,596	2,958,932	77.1
Continuity of Care - PO	351	460,022	87.5	3,596	2,958,932	87.5
Diabetes Eye Exams	121	38,321	58.8	1,050	240,835	58.5
Follow-up after Acute Events	55	11,709	67.1	362	55,608	66.4
Hypertension Medication Adherence	271	175,512	83.3	2,538	1,089,560	81.7
Statin Prescription for CVD	85	24,181	68.3	631	135,193	67.5
**Utilization measures**						
PCC visits per 100 beneficiaries, mean	442	744,597	437.3	4,499	4,621,417	468.7
IM specialist visits per 100 beneficiaries, mean	442	744,597	195.6	4,499	4,621,417	201.2
Ophthalmologist/optometrist visits per 100 beneficiaries, mean	442	744,597	117.7	4,499	4,621,417	116.3
ED visits per 100 beneficiaries, mean	442	744,597	36.8	4,499	4,621,417	35.5
Ambulatory care-sensitive ED visits/hospitalizations per 100 beneficiaries, mean	442	744,597	7.3	4,499	4,621,417	7.2

Abbreviations: PO, physician organization; PCC, primary care clinician; CVD, cardiovascular disease; ED, emergency department; IM, internal medicine.

[a] All POs included in the analysis met all inclusion criteria defined in eTable A1 and had at least 30 beneficiaries eligible for the measure of interest in each of the 7 study years. Beneficiary counts and performance are measured in the baseline year, 2013.

© 2025 Timbie JW et al. *JAMA Network Open.*

eTable 3. Quality and Utilization Measure Specifications

**Measure**	**Description**
All-Cause Hospital Readmissions	Indicator of an unplanned readmission for any reason within 30 days of discharge from the hospital. We used the National Committee for Quality Assurance's HEDIS measure specifications for Plan All-Cause Readmissions for measurement years 2013-2019. Beneficiaries are eligible for the measure if they are age 18 and older and are discharged alive from the hospital. The unit of analysis is the hospital discharge. A hospital discharge is identified as having a readmission if the beneficiary is readmitted to a hospital within 30 days, either for the condition associated with the index hospital stay or for a different reason and excludes planned readmissions such as for chemotherapy. The measure specifications include case mix adjustment using a beneficiary's age, gender, primary discharge diagnosis, comorbidities, and other factors.
Breast Cancer Screening	Indicator for receipt of a mammogram during the measurement year or the preceding 15 months. We used the National Committee for Quality Assurance's HEDIS measure specifications for Breast Cancer Screening for measurement years 2013-2019. The measure assesses whether female Medicare beneficiaries aged 50–74 receive a mammogram to screen for breast cancer during the measurement year or the preceding 15 months. Women with a diagnosis of bilateral mastectomy in their claims history are excluded from the measure.
Continuity of Care – PCC[a]	Continuous measure of the proportion of primary care visits that were made by a beneficiary to his or her usual source of primary care during the measurement year. A beneficiary's usual source of primary care was defined as the primary care clinician associated with a plurality of the beneficiary's primary care visits. Primary care clinicians were defined as those who reported one of the following 8 specialties in the MDPPAS file: family medicine, general internal medicine, general pediatrics, general practice, nurse practitioner, physician assistant, geriatrics, and gynecology. Eligible primary care clinicians were those whose first "primary specialty" listed in MDPPAS was one of the 8 specialties listed above and who either had no second "primary specialty" or whose second "primary specialty" was also included in the list above. Beneficiaries were eligible for this measure if they had three or more primary care visits during the measurement year. The denominator for the measure is the total count of primary care visits for the beneficiary in the measurement year based on the specifications for the measure *Primary Care Visits* (see below). The numerator was the number of these visits that were made to the beneficiary's usual source of care.
Continuity of Care – PO[a]	Continuous measure of the proportion of primary care visits that were made by a beneficiary to the *physician organization* that represents the

© 2025 Timbie JW et al. *JAMA Network Open.*

Measure	Description
	beneficiary's usual source of primary care during the measurement year. The measure is analogous to *Continuity of Care – PCC* but aggregates counts of primary care visits to the physician organization level rather than to the level of individual clinicians.
Diabetes Eye Exam	Indicator for receipt of an eye screening for diabetic retinal disease during the measurement year among beneficiaries with Type 1 or Type 2 diabetes. We used the National Committee for Quality Assurance's HEDIS measure specifications for the measure *Comprehensive Diabetes Care* for measurement years 2013-2019. The measure assesses whether beneficiaries age 18–75 with Type 1 or Type 2 diabetes received an eye screening for diabetic retinal disease during the measurement year. The eye exam must be performed by an eye care professional (i.e., optometrist or ophthalmologist). Alternatively, an eye exam provided by an eye care professional in the prior year with a negative test result for retinopathy would also qualify as a success for the measure. Beneficiaries who had a diagnosis of gestational diabetes or steroid-induced diabetes during the measurement year are excluded from the measure.
Follow-up after Acute Events	Indicator of timely follow-up after acute events. We use the specifications for the measure *Timely Follow-up after Acute Exacerbations of Chronic Conditions*, developed by IMPAQ International, LLC.[1] This measure assesses timely follow-up care for beneficiaries who have one of 6 chronic conditions: hypertension, asthma, heart failure, coronary artery disease, chronic obstructive pulmonary disease, or diabetes mellitus, and who experience acute events identified by diagnosis codes and utilization in one of three settings: emergency department, observation unit, or inpatient hospital. Follow-up visits are defined as "timely" if they occur within 7 days (hypertension), 14 days (asthma, heart failure, and CAD), or within 30 days (COPD and diabetes).
Hypertension Medication Adherence	Indicator for a beneficiary who adheres to his or her prescribed drug therapy for renin angiotensin system (RAS) antagonists: angiotensin converting enzyme inhibitor (ACEI), angiotensin receptor blocker (ARB), or direct renin inhibitor medications. We used specifications from the Medicare 2017 Part C & D Star Ratings[2] which are endorsed by the National Quality Forum (NQF) and the Pharmacy Quality Alliance (PQA). Beneficiaries are eligible for this

[1] Martin B, Shirley B, Chiu J, Pedersen S. Implementation Plan for the Medicare Advantage Care Coordination Measure Set.

[2] Centers for Medicare & Medicaid Services. Medicare 2017 Part C & D Star Rating Technical Notes. Accessed April 15, 2020, https://www.cms.gov/Medicare/Prescription-Drug-Coverage/PrescriptionDrugCovGenIn/Downloads/2017_Technical_Notes_preview_1_2016_08_03.pdf

© 2025 Timbie JW et al. *JAMA Network Open.*

Measure	Description
	measure if they are age 18 years and older and filled prescriptions for at least two RAS antagonists on unique dates of service during the measurement year. Adherence is defined as a sufficient number of prescription fills covering 80% or more of the time the beneficiary is supposed to be taking the medication (i.e., proportion of days covered at 80 percent or higher).
Statin Prescription for Cardiovascular Disease	Indicator for dispensing of statin medication during the measurement year for beneficiaries with diabetes. We used the National Committee for Quality Assurance's HEDIS measure specifications for *Statin Therapy for Patients With Cardiovascular Disease* for measurement years 2017-2019. We use the indicator: *Received Statin Therapy,* which identifies beneficiaries who were dispensed at least one high-intensity or moderate-intensity statin medication during the measurement year.<br>Beneficiaries are eligible for the measure if they are males 21–75 years of age and females 40–75 years of age during the measurement year, and who have clinical atherosclerotic cardiovascular disease.
Primary care clinician visits[a]	Count of face-to-face encounters with a physician reporting one of the following 8 specialties in the MDPPAS file: family medicine, general internal medicine, general pediatrics, general practice, nurse practitioner, physician assistant, geriatrics, and gynecology. Eligible primary care providers were those whose first "primary specialty" from MD_PPAS was one of the 8 specialties listed above and who either had no second "primary specialty" or whose second "primary specialty" was also included in the list above. Visits were defined using HCPCS codes for evaluation and management services. Facility-based providers (i.e., critical access hospitals, rural health clinics, federally qualified health centers, Electing Teaching Amendment ("ETA") hospitals) that provide evaluation and management services were identified using specific revenue center codes and facility type fields on outpatient claims.
Internal medicine specialist visits[a]	Count of face-to-face encounters with a physician who reported one of the following 11 internal medicine sub-specialties as his or her "primary specialty" in the MDPPAS file: cardiology, critical care medicine, endocrinology, gastroenterology, hematology, immunology, infectious disease, nephrology, oncology, pulmonary disease, and rheumatology. Visits were defined using HCPCS codes for evaluation and management services. Facility-based providers (i.e., critical access hospitals, rural health clinics, federally qualified health centers, Electing Teaching Amendment ("ETA") hospitals) that provide evaluation and management services were identified using specific revenue center codes and facility type fields on outpatient claims.

© 2025 Timbie JW et al. *JAMA Network Open.*

Measure	Description
Ophthalmologist/optometrist visits[a]	Count of visits to ophthalmologists or optometrists. Encounters were identified using evaluation and management procedure codes or codes corresponding to one of 16 eye exam procedures. Eye doctors were identified as those whose "primary specialty" in the MDPPAS file was either ophthalmologist or optometrist.
Emergency department visits[a]	Count of visits to the Emergency Department for any reason during the measurement year. Visits are identified by revenue center codes and CPT codes. ED visits include both visits that do and do not lead to admission.
Ambulatory care-sensitive ED visits and hospitalizations[a]	Count of ambulatory-care sensitive visits to acute care settings during the measurement year. We used the AHRQ Prevention Quality Indicators[3] to identify ambulatory-care sensitive hospitalizations, and we applied these specifications to Emergency Department claims to identify ACS-ED visits following an approach used previously by others.[4] The 8 indicators comprising the measure include hospitalizations or ED visits for: diabetes short-term complications (PQI 01); diabetes long-term complications (PQI 03); chronic obstructive pulmonary disease or asthma in older adults (PQI 05); hypertension (PQI 07); heart failure (PQI 08); community-acquired pneumonia (PQI 11); urinary tract infection (PQI 12); and uncontrolled diabetes (PQI 14).

[a] Beneficiaries were eligible for these measures if they were age 18 and older, had 12 months of Medicare entitlement in the measurement year, had no months of Medicare Advantage enrollment, were alive at the end of the measurement year, were not entitled to Medicare coverage because of End-Stage Renal Disease (ESRD), and had no hospice use in the measurement year.

---

[3] Agency for Healthcare Research and Quality. PQI Technical Documentation. https://qualityindicators.ahrq.gov/measures/PQI_TechSpec

[4] Feng Z, Silver B, Segelman M, et al. Developing Risk-Adjusted Avoidable Hospitalizations and Emergency Department Visits Quality Measures: Final Report. 2019. https://www.medpac.gov/wp-content/uploads/import_data/scrape_files/docs/default-source/contractor-reports/august2019_riskadjusted_ah_av_measures_contractor_sec.pdf

© 2025 Timbie JW et al. *JAMA Network Open.*

eTable 4. Parallel Trends Assessment

	Difference-in-Difference Estimate Relative to Year -1[a]					
	Year -4 and -5		Year -3		Year -2	
Measure	Estimate	P Value	Estimate	P Value	Estimate	P Value
Quality measures						
All-cause Hospital Readmissions, %	0.1	0.906	0.1	0.905	-0.9	0.109
Breast Cancer Screening, %	-1.0	0.264	-0.1	0.947	-0.5	0.567
Continuity of Care – PCC, %	-0.4	0.104	-0.1	0.832	0.1	0.701
Continuity of Care – PO, %	-0.4	0.089	-0.2	0.435	-0.1	0.620
Diabetes Eye Exams, %	1.6	0.159	1.3	0.300	2.1	0.073
Follow-up after Acute Events, %	-2.0	0.232	-1.0	0.612	0.0	0.982
Hypertension Med. Adherence, %	-0.5	0.360	0.1	0.873	-0.5	0.358
Statin Prescription for CVD, %	0.1	0.950	0.4	0.745	0.6	0.613
Utilization measures						
PCC visits per beneficiary, mean	0.002	0.968	-0.026	0.618	-0.017	0.738
IM specialist visits per beneficiary, mean	0.000	0.999	0.029	0.396	0.015	0.640
Ophthalmologist/optometrist visits per beneficiary, mean	0.006	0.697	0.009	0.610	-0.017	0.283
ED visits per beneficiary, mean	0.004	0.757	0.005	0.698	0.011	0.349
Ambulatory care-sensitive ED visits/hospitalizations per beneficiary, mean	0.008	0.123	0.007	0.234	0.009	0.089

Abbreviations: PO, physician organization; PCC, primary care clinician; CVD, cardiovascular disease; ED, emergency department; IM, internal medicine.

[a] All estimates are difference-in-difference-in-differences estimates that estimate changes in within-PO disparities for duals relative to non-duals in POs that undergo affiliation relative to those that do not in each pre-affiliation year relative to the year prior to a PO's affiliation with a health system ("Year -1"). Statistically significant estimates indicate a departure from parallel trends in within-PO disparities between POs that affiliate and those that do not.

© 2025 Timbie JW et al. *JAMA Network Open.*

eTable 5. Overall Disparities in Quality of Care Between Dual-Eligible and Non–Dual-Eligible Beneficiaries Before Affiliation and Impact Of Affiliation (Figure 1 in Manuscript)

	**Pre-Affiliation Disparity**		**Impact of Affiliation on Disparity**		**Impact of Affiliation by Group**	
					**Dual-eligible**	**Non–dual-eligible**
**Quality Measure**	**Estimate**	**SE**	**Estimate**	**SE**	**Estimate**	**Estimate**
Breast Cancer Screening	-0.131	0.002	-0.010	0.003	-0.011	-0.001
Diabetes Eye Exams	-0.059	0.002	-0.035	0.005	-0.017	0.018
Follow-up after Acute Events	-0.055	0.002	-0.035	0.008	-0.036	-0.001
Hypertension Medication Adherence	-0.011	0.001	0.004	0.002	0.003	-0.001
All-cause Hospital Readmissions	0.011	0.000	0.007	0.002	0.009	0.002
Statin Prescription for CVD	0.012	0.002	0.018	0.006	0.020	0.002
Continuity of Care – PCC	0.016	0.000	0.019	0.001	0.007	-0.012
Continuity of Care – PO	0.019	0.000	0.014	0.001	0.024	0.010

Abbreviations: PO, physician organization; PCC, primary care clinician; CVD, cardiovascular disease.

© 2025 Timbie JW et al. *JAMA Network Open.*

eTable 6. Overall Disparities in Utilization Between Dual-Eligible and Non–Dual-Eligible Beneficiaries Before Affiliation and Impact of Affiliation (Figure 2 in Manuscript)

	Pre-Affiliation Disparity		Impact of Affiliation on Disparity		Impact of Affiliation by Group	
					Dual-eligible	Non–dual-eligible
**Utilization Measure**	**Estimate**	**SE**	**Estimate**	**SE**	**Estimate**	**Estimate**
Internal medicine specialist visits per beneficiary	-0.888	0.005	-0.028	0.009	-0.064	-0.036
PCC visits per beneficiary	-0.378	0.006	-0.208	0.011	-0.271	-0.063
Ophthalmologist/optometrist visits per beneficiary	-0.186	0.003	-0.050	0.005	-0.046	0.004
Ambulatory care-sensitive acute care visits per beneficiary	0.031	0.000	0.008	0.001	0.008	0.000
ED visits per beneficiary	0.156	0.001	0.014	0.003	0.010	-0.004

Abbreviations: PCC, primary care clinician; ED, emergency department.

© 2025 Timbie JW et al. *JAMA Network Open.*

eTable 7. Within-PO and Between-PO Disparities in Quality of Care Between Dual-Eligible and Non–Dual-Eligible Beneficiaries Before Affiliation and Impact of Affiliation (Figure 3 in Manuscript)

	**Within-PO Disparity**				**Between-PO Disparity**			
	**Pre-Affiliation Disparity**		**Impact of Affiliation on Disparity**		**Pre-Affiliation Disparity**		**Impact of Affiliation on Disparity**	
**Quality Measure**	**Estimate**	**SE**	**Estimate**	**SE**	**Estimate**	**SE**	**Estimate**	**SE**
Breast Cancer Screening	-0.122	0.001	-0.009	0.003	-0.052	0.002	-0.004	0.004
Diabetes Eye Exams	-0.061	0.001	-0.030	0.004	-0.016	0.003	-0.016	0.005
Follow-up after Acute Events	-0.052	0.002	-0.023	0.007	-0.025	0.005	-0.037	0.013
Hypertension Medication Adherence	-0.002	0.000	-0.002	0.002	-0.014	0.001	0.008	0.002
All-cause Hospital Readmissions	0.008	0.000	0.005	0.002	0.007	0.001	0.007	0.002
Statin Prescription for CVD	0.017	0.001	0.012	0.005	-0.003	0.003	0.014	0.008
Continuity of Care – PCC	0.022	0.000	0.004	0.001	-0.004	0.001	0.025	0.001
Continuity of Care – PO	0.023	0.000	0.009	0.001	0.001	0.001	0.012	0.001

Abbreviations: PO, physician organization; PCC, primary care clinician; CVD, cardiovascular disease.

© 2025 Timbie JW et al. *JAMA Network Open.*

eTable 8. Within-PO and Between-PO Disparities in Utilization Between Dual-Eligible and Non–Dual-Eligible Beneficiaries Before Affiliation and Impact of Affiliation (Figure 4 in Manuscript)

	**Within-PO Disparity**				**Between-PO Disparity**			
	**Pre-Affiliation Disparity**		**Impact of Affiliation on Disparity**		**Pre-Affiliation Disparity**		**Impact of Affiliation on Disparity**	
**Utilization Measure**	**Estimate**	**SE**	**Estimate**	**SE**	**Estimate**	**SE**	**Estimate**	**SE**
Internal medicine specialist visits per beneficiary	-0.862	0.002	-0.066	0.008	-0.302	0.008	0.046	0.011
PCC visits per beneficiary	-0.190	0.002	-0.200	0.010	-0.374	0.010	-0.074	0.013
Ophthalmologist/optometrist visits per beneficiary	-0.199	0.001	-0.047	0.005	-0.039	0.004	-0.020	0.006
Ambulatory care-sensitive acute care visits per beneficiary	0.029	0.000	0.009	0.001	0.011	0.001	0.000	0.001
ED visits per beneficiary	0.153	0.000	0.019	0.002	0.052	0.002	-0.003	0.003

Abbreviations: PO, physician organization; PCC, primary care clinician; ED, emergency department.

© 2025 Timbie JW et al. *JAMA Network Open.*

eTable 9. Comparisons of Beneficiary Characteristics by Dual-Eligibility Status and Attributed PO Type

	Becomes Affiliated[a]		Never Affiliated	
	Dual-eligible	Non–dual-eligible	Dual-eligible	Non–dual-eligible
Characteristic	Patients, No. (%)	Patients, No. (%)	Patients, No. (%)	Patients, No. (%)
Age, mean (SD), y	76.3 (8.0)	75.7 (7.6)	76.0 (7.9)	75.4 (7.5)
Female	71.5	57.3	69.6	56.7
Disabled	24.4	6.7	22.6	7.1
Race/ethnicity				
Asian/Pacific Islander	5.0	0.8	7.0	1.1
Black	15.9	4.6	16.2	5.6
Hispanic	10.3	1.8	18.3	2.9
White	66.6	91.3	54.7	88.6
Other[b]	1.3	0.7	3.0	1.2
Urbanicity				
Metropolitan RUCA[c]	73.9	77.8	74.1	78.4
Micropolitan RUCA	13.7	12.0	13.3	12.1
Small Town RUCA	7.6	6.2	7.5	5.6
Rural RUCA	4.7	3.9	5.0	3.8
HCC Score, mean (SD)[d]	1.6 (1.2)	1.0 (0.9)	1.6 (1.2)	1.0 (0.9)
Area-level SES Index, mean (SD)[e]	-0.4 (1.0)	0.2 (0.8)	-0.5 (1.0)	0.1 (0.9)

[a] Characteristics of 4,268 POs meeting all inclusion criteria for the analysis are displayed in the table and reflect values in the baseline year (2013).

[b] This group includes American Indian/Alaska Native beneficiaries and beneficiaries with “Other” race listed in the Medicare Beneficiary Summary File.

[c] RUCA=Rural-Urban Commuting Area

[d] HCC=Hierarchical Condition Category

[e] The area-level Socioeconomic Status (SES) index includes 6 items: percent graduating high school, percent male unemployment, percent of households living below poverty, percent of female-headed households with children, percent of households receiving public assistance, and median annual household income.

© 2025 Timbie JW et al. *JAMA Network Open.*

eFigure. Impact of Health System Affiliation for Dual-Eligible and Non–Dual-Eligible Beneficiaries Who Receive Care From POs With a Low or High Percentage of Dual-Eligible Beneficiaries

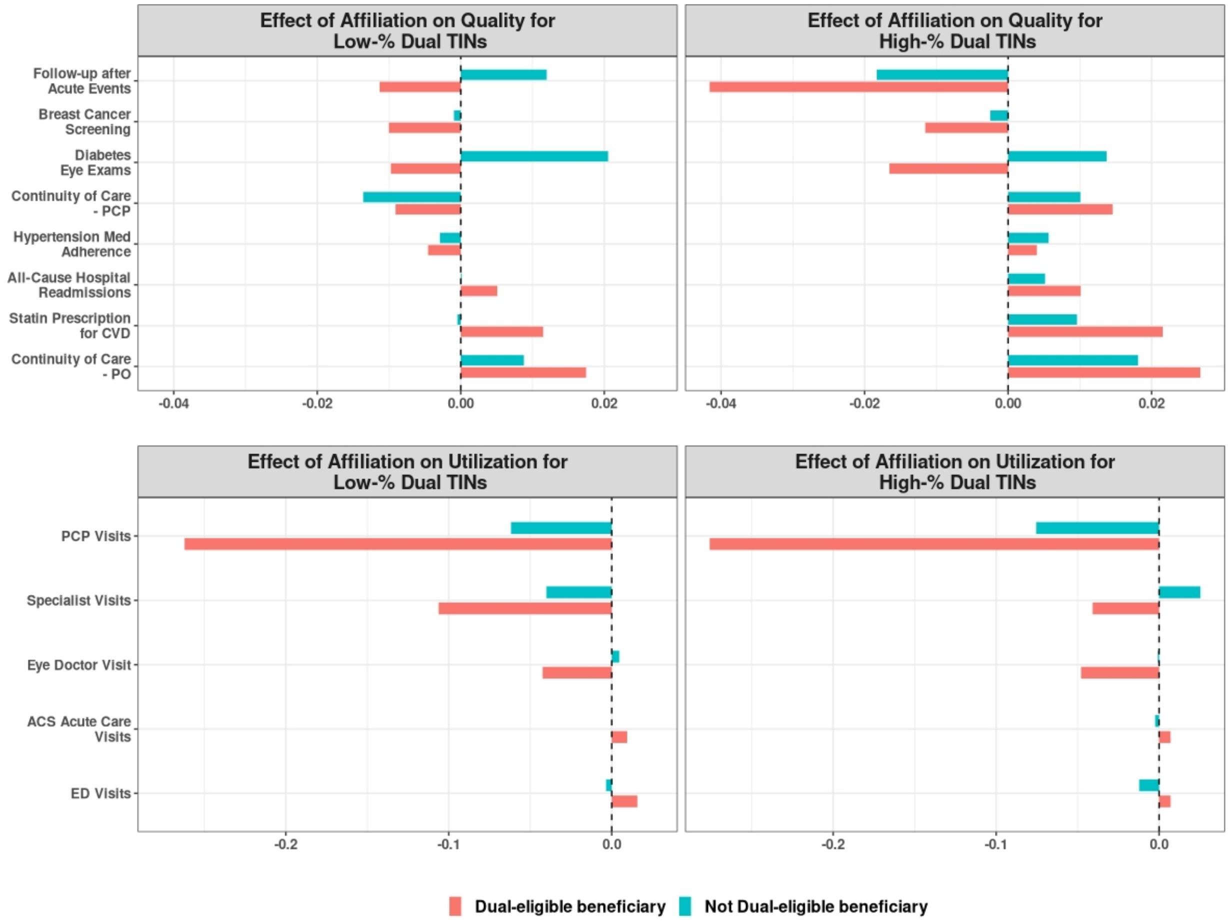

© 2025 Timbie JW et al. *JAMA Network Open.*

eMethods.

1. Identifying newly affiliating POs

Identifying new health system affiliations is challenging when using TINs as a PO identifier because affiliating POs commonly adopt the TIN of their health system leading to a "censoring" problem for these TINs in Medicare FFS claims data. To address this issue, we first identified POs for which the majority of physicians billing with a TIN in one calendar year begin using a different TIN in the next year and for which the original TIN never appears in subsequent years in the MDPPAS. These TIN changes could reflect either a consolidation between organizations or simply a change in the use of a TIN without an actual consolidation. For these POs, we refer to the PO's original TIN as its "legacy" TIN and the new TIN as the "surviving" TIN, and we update the TIN listed for each provider in the MDPPAS (i.e., surviving TIN) with the provider's legacy TIN. We refer to this version of the MDPPAS file as the "updated MDPPAS," and we carried forward the legacy TIN in each subsequent year of the updated MDPPAS so long as the provider continued to bill Medicare Part B services most commonly using the surviving TIN listed in the original MDPPAS.

We used both the "original MDPPAS" and "updated MDPPAS" to attribute beneficiaries to POs. We first attributed beneficiaries to POs based on TINs listed in the original MDPPAS file as described in the Methods section. We then used the updated MDPPAS to update PO attributions for beneficiaries attributed to a surviving TIN to determine whether to attribute the beneficiary the legacy TIN or surviving TIN based on the plurality of E&M visits to providers associated with each TIN.

2. Statistical model for decomposing impacts of health system affiliation on within-PO and between-PO disparities

Drawing on prior studies that distinguish within- and between-unit effects in multilevel or hierarchical data structures (Bell et al. (2019) and Hamaker and Muthen (2020)), we use a single statistical model to estimate all quantities of interest. Let y_{ijt} be a quality or utilization measure for Medicare beneficiary i, attributed to PO j, in year t, where $i = 1,2,\dots,n_{jt}$, $j = 1,2,\dots,g_t$, and $t = 1,2,\dots,T$. Let x_{ijt} be an individual-level characteristic that defines the disparity of interest, such as an indicator of dual eligibility. Let z_{jt} be a group-time level characteristic, such as the affiliation status of PO j in year t. No POs are considered to be affiliated in the year of affiliation; the first post-affiliation measurement year is defined as the first full calendar year after affiliation. To facilitate notation, we define $I_{ijt} = x_{ijt}z_{jt}$, an interaction between the beneficiary-level characteristic and the group-time level characteristic. We use the following multi-level linear model:

$$y_{ijt} = \beta_0 + \beta_{w1}(x_{ijt} - \bar{x}_{jt}) + \beta_{b1}\bar{x}_{jt} + \beta_{w2}(I_{ijt} - \bar{I}_{jt}) + \beta_{b2}\bar{I}_{jt} + \beta_{w3}(z_{jt} - \bar{z}_j) + \beta_{b3}\bar{z}_j + \delta_t + u_j + u_{jt} + e_{ijt}$$

© 2025 Timbie JW et al. *JAMA Network Open.*

Here δ_t are year fixed effects, with $\delta_1 = 0$, $u_j \sim N(0, \sigma_{u1}^2)$ are random intercepts for PO, $u_{jt} \sim N(0, \sigma_{u2}^2)$ are random intercepts for PO-year, and $e_{ijt} \sim N(0, \sigma_e^2)$ is an error term. (Note: for some outcomes, including PO-year random intercepts led to convergence problems or singularities, so just PO random intercepts were included.)

The interpretations of key coefficients are as follows:

- β_{w1}: Pre-affiliation (or unaffiliated) within-PO disparity (dual vs. non-dual)
- β_{b1}: Pre-affiliation between-PO disparity
- β_{w2}: Effect of affiliation on within-PO disparity
- β_{b2}: Effect of affiliation on between-PO disparity.

Other coefficients that are not central to the decomposition results include:

- β_{w3}: Effect of within-PO affiliation changes for non-duals in 0% dual POs
- β_{b3}: Effect of enduring cross-PO differences in affiliation status.

In **Figures 1 and 2 of the manuscript**, we report "overall" disparity estimates both before and after affiliation that account for both within-PO disparities and between-PO disparities. These estimates are derived as follows:

- Panel 1: Pre-affiliation disparity: $r*\beta_{b1} + (1-r)*\beta_{w1}$
- Panel 2: Effect of affiliation on disparity: $r*\beta_{b2} + (1-r)*\beta_{w2}$
- Panel 3: Effect of affiliation on disparity by group:
  - Dual: $(\beta_{b2} - \beta_{w2})*a + \beta_{w2} + \beta_{w3}$
  - Non-dual: $(\beta_{b2} - \beta_{w2})*b + \beta_{w3}$

In the formulas above, *a* is the mean PO-level percentage dual among dual-eligible beneficiaries across all years; *b* is the mean PO-level percentage dual among non-duals across all years; and *r*=(*a*-*b*).

In **Figures 3 and 4 of the manuscript**, we report the within-PO disparity estimates directly from the model (i.e., β_{w1} and β_{w2}). In both figures we rescale the between-PO disparity estimates (i.e., β_{b1} and β_{b2}) to reflect POs at the 20th and 80th percentiles of the PO-level distribution of percent dual.

© 2025 Timbie JW et al. *JAMA Network Open.*

eReferences.

DeYoreo M, Agniel D, Zhang SY, Escarce JJ, Timbie JW. Estimating an intervention's effects on health care disparities within and between physician organizations: decomposing the effects of health system affiliation. Health Serv Outcome. Jun 14 2025;doi:10.1007/s10742-025-00350-z

Bell A, Fairbrother M, Jones K. Fixed and random effects models: Making an informed choice. Quality & Quantity: International Journal of Methodology. 2019;53(2):1051–1074.

Hamaker EL, Muthen B. The fixed versus random effects debate and how it relates to centering in multilevel modeling. Psychol Methods. Jun 2020;25(3):365-379. doi:10.1037/met0000239

© 2025 Timbie JW et al. *JAMA Network Open.*
