# Supplementary material for: Health System Affiliation and Care for Dual-Eligible and Non–Dual-Eligible Medicare Beneficiaries
Source: JAMA Netw Open. 2025 Oct 23;8(10):e2538770. doi: 10.1001/jamanetworkopen.2025.38770 (PMC12550639; doi:10.1001/jamanetworkopen.2025.38770)
Supplement: Supplement 2. — Data Sharing Statement [file jamanetwopen-e2538770-s002.pdf]

Data Sharing Statement

Timbie. Health System Affiliation and Care for Dual-Eligible and Non–Dual-Eligible Medicare Beneficiaries. *JAMA Netw Open*. Published October 23, 2025.
doi:10.1001/jamanetworkopen.2025.38770

Data
Data available: No

Additional Information
Explanation for why data not available: My CMS DUA precludes data sharing
